# Psychological and Occupational Factors Associated with Depression Among Healthcare Providers During the COVID – 19 Pandemic in A Regional Referral Hospital in Kisumu County

**DOI:** 10.24248/eahrj.v8i3.798

**Published:** 2025-01-30

**Authors:** Jared Makori Bundi, Moses Poipoi, Everlyne Nyanchera Morema

**Affiliations:** a Department of Community Health Nursing, School of Nursing, Maseno University, Maseno, Kenya; b Department of Educational Psychology, School of Education, Masinde Muliro University of Science and Technology (MMUST), Kakamega, Kenya; c Department of Community Health Nursing, School of Nursing, Midwifery and Paramedical Sciences (SONMAPS), Masinde Muliro University of Science and Technology (MMUST) Kakamega, Kenya.

## Abstract

**Background:**

Corona Virus Disease of 2019 (COVID-19) spread across the globe, causing distress among various populations, including healthcare providers. This disease has had an unparalleled effect on the world’s economic situation, livelihood, mental and physical well-being across the globe.

The study aimed to determine the prevalence of depression and identify the occupational and psychological factors associated with depression among health care providers during the COVID-19 pandemic in a regional referral hospital in Kisumu County.

**Methods:**

We conducted a hospital-based cross-sectional study at JOOTRH where a total of 202 respondents participated in the study. The survey questionnaire consisted of four components: demographic factors, occupational factors, and psychological factors. Depression was measured using the 9-item Patient Health Questionnaire (PHQ-9). Data was analysed using the statistical package for Social Science version 28. Pearson chi-square was used to determine the occupational and psychological factors associated with depression during the COVID-19 pandemic at *p ≤.05*.

**Results:**

The overall prevalence of depression was at 57.4%. The occupational and psychological factors associated with depression among healthcare providers during the COVID-19 pandemic included being over 30 years old, married, having over 6 years of work experience, COVID-19 training, having an irregular work schedule, lacking psychological support services, and facing stigma.

**Conclusion:**

The study findings revealed a considerable proportion of depressive symptoms among health providers during the COVID-19 pandemic at JOOTRH. Older age, being married, more years of work experience, previous exposure to pandemic, having COVID-19 training, and irregular work schedule, experience of stigma, and lack of psychological support were significantly associated with depression.

## BACKGROUND

Corona Virus Disease of 2019 (COVID-19) was the sixth health crisis of public health importance globally, caused by Severe Acute Respiratory Syndrome Coronavirus–2.^1,2^ Emerging psychiatric morbidities and mental well-being were identified as the tenth most frequent research topic during the COVID–19 pandemic. ^3^ Multiple studies, including systematic reviews, established relatively high rates of symptoms of anxiety, depression, post-traumatic stress disorder, and stress in the general population and health care professionals during the COVID-19 pandemic globally.^4^ Among the mental disorders, depression was ranked as the most common, with approximately 210 million people affected globally, with Africa having the highest number of cases. In Africa, Kenya is ranked fourth, with approximately 1.9 million people having depression.^5^

Many African countries, including Kenya, grappled with the rising cases of the COVID-19 pandemic in the midst of insufficient medical resources and infrastructure, systemic challenges, challenges in the implementation of key policies set by these countries, and inadequate health care personnel to adequately address the COVID-19 pandemic.^6^ Kisumu County, where the regional referral hospital is located, has a myriad of challenges, with the County’s Integrated Development Plan (CIDP) depicting health care provider-to-population ratios that were unfavourable and continued on a worsening trajectory as a result of the pandemic and its attendant austerity measures. The health workforce is severely stretched in terms of number, capacity, and mental resilience. The problem is further compounded by a high prevalence of infectious and non-communicable diseases and the fact that the county has no well-laid formal mental health care plan for the caregivers within the COVID-19 response strategy.^7,8^ The current study, therefore, was to determine the prevalence of depression and associated factors among health care providers during the COVID-19 pandemic at a regional teaching and referral hospital in Western Kenya.

## METHODS

### Study Design and Study Population

This was a descriptive cross-sectional study targeting 352 health care providers who had been actively involved in treatment and care of patients during the pandemic in the regional teaching and referral hospital.

### Sampling Techniques

This study used proportionate sampling with the sample size for each cadre determined by their proportion in the target population. A sampling frame was created based on the staff establishment, inputted into the excel worksheet and random numbers were generated and assigned to this frame. These numbers were then sorted in an ascending order. From this ordered list, the sample size for each cadre was drawn. To ensure a 100% response rate, if a selected respondent declined to participate, the next respondent in the sorted list was approached. The process was repeated for each cadre to maintain the proportionate representation in the sample.

### Methods and Instruments

The study used a self-administered Kobo Toolbox-based questionnaire during the COVID-19 pandemic. The questionnaire had the demographic, occupational, and psychological characteristics of health care providers. Depression of health care providers was measured by the 9-item Patient Health Questionnaire (PHQ-9). The validated tool was previously used in research related to the COVID-19 pandemic.^9,10^

The questionnaire has nine items, with each item assigned scores of 0, 1, 2, and 3, representing not at all, several days, more than half the days, and nearly every day, respectively. The total score that was generated from the tool is 27, with score ranges of 1 to 4, 5 to 9, 10 to 14, 15 to 19, and 20 to 27 representing minimal, mild, moderate, moderately severe, and severe depression, respectively. The nine items over the past 2 weeks are little interest or pleasure in doing things, depressed, feeling down or hopeless, insomnia or hypersomnia, fatigue or having little energy, poor appetite or overeating, guilt or worthlessness, trouble concentrating, psychomotor agitation or retardation, and suicidal thoughts or ideas. The tool has been validated for use in primary care, with aspects of its construct validity documented in studies both in the general population and medical settings.^11^

### Sample Size Calculation

The sample size was estimated based on Fisher and Laing (1988), n = Z^2^pq/d^2^ Where n is the desired sample size (when study target population is over 10,000), Z–Is the standard normal deviate = 1.96 (corresponding to 95% Confidence Interval), p–Proportion of the target population estimated to have a particular characteristic. If there is no reasonable estimate then use 50 percent, therefore *P =.50*. q = 1.0-p = 1–0.5 = 0.5, d = Degree of accuracy (Margin of error) desired usually set as 0.05.^12^ Hence the desired sample size (n) will be calculated as follows. n = 1.962 × 0.5 × 0.5/(0.05)2. Thus n = 384.16.

Since the target population is less than 10,000 the sample size is adjusted using the Cochran formula for finite population nf = n/1+ (n/N). Where nf = desired sample size when the population is finite and less than 10,000, n = the desired sample size when the population is more than 10,000, N = estimated population size, nf = 384/1+ (384/352), nf = 184. Therefore 10% will be added to take care of spoilt questionnaires and the non-responses; 10% of 184 = 18, thus 184+18 = 202. A sample population of 202 health care providers was generated, inclusive of the margin for nonresponse, and included in the study.

### Statistical Analysis

Data was exported from the Kobo Collect platform in Excel format, cleaned, and exported to Statistical Package for Social Sciences version 28 for analysis (SPSS). The data collected was analysed using descriptive and inferential statistics. Bivariate logistic regression was used to examine the occupational and psychological factors associated with depression during the COVID-19 pandemic, and odds ratio was used to assess the strength of the association. All the statistical tests were performed at a 0.05 level of significance (95% confidence interval).

### Ethics Approval

The study was approved by Masinde Muliro University of Science and Technology Institutional Scientific and Ethics Review Committee (MMUST-ISERC) approval number MMUST/IERC/062/2022 and Jaramogi Oginga Odinga Teaching and Referral Hospital Institutional Scientific Ethical Committee (JOOTRH-ISERC) approval number IERC/JOOTRH/619/22. The researchers also obtained a research license from The National Commission of Science, Technology, and Innovation (NACOSTI) license number NACOSTI/P/22/18058. Considerable time was taken to address ethical principles relating to beneficence and non-maleficence, anonymity and confidentiality, justice, and autonomy. After the conclusion of the study, a score for depression was computed, and if the score was indicative of clinically significant depression, they were directed for further evaluation and assistance.

## RESULTS

### Levels of Depression among Health Care Providers

In this study, a good number of health providers (14.4%) reported feeling down, depressed, or hopelessness nearly every day and 13.4% reported feeling tired or having little energy. The findings on each item on the PHQ - 9 scale are further described in Table 1.

**Table 1:** Distribution of Depression Related Aspects on the PHQ - 9 Scale

Variable on the PHQ-9 Scale	Not at all	Several days	More than half the days	Nearly everyday
Little interest or pleasure in doing things	53 (26.2)	55 (27.2)	73 (36.1)	21 (10.4)
Feeling down, depressed, or hopeless	43 (21.3)	105 (52)	25 (12.4)	29 (14.4)
Trouble falling or staying asleep, or sleeping too much	55 (27.2)	66 (32.7)	64 (31.7)	17 (8.4)
Feeling tired or having little energy	19 (9.4)	78 (38.6)	78 (38.6)	27 (13.4)
Poor appetite or overeating	33 (16.3)	56 (27.7)	87 (43.1)	26 (12.9)
Feeling bad about self for instance failure or let down to self or family	58 (28.7)	106 (52.5)	17 (8.4)	21 (10.4)
Trouble concentrating	56 (27.7)	66 (32.7)	55 (27.2)	25 (12.4)
Moving or speaking noticeably slowly or fidgety or restless or moving around a lot more than usual	74 (36.6)	74 (36.6)	37 (18.3)	17 (8.4)
Having suicidal thoughts or self-harm	137 (67.8)	38 (18.8)	16 (7.9)	11 (5.4)

The PHQ-9 scale grades the frequency of the feelings experienced by the respondent as ranging from 0 (for not at all) to 3 (nearly every day). The maximum grades for each of the 9 items add up to a maximum score of 27, which is considered the highest measure for depression. To describe the levels of depression, the scores were further categorised into 4. Scores of 0 to 4 represented minimum depression, 5 to 9 mild depression, 10 to 14 moderate depression, 15 to 19 moderately severe depression, and 20 to 27 as severe depression.

**Figure 1.**
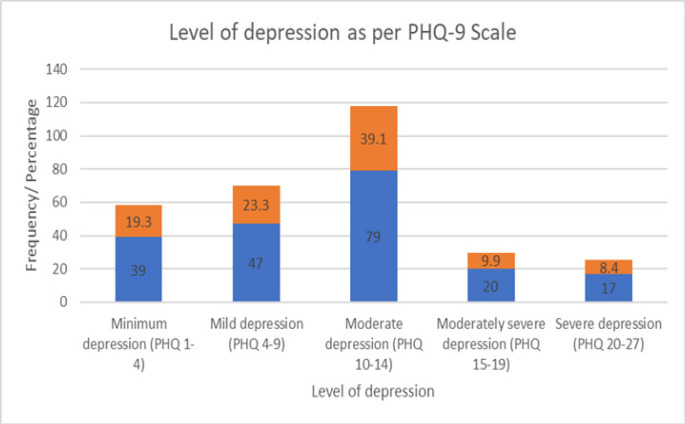
Levels of Depression Measured Using PHQ-9 Scale

The level of minimum depression among the respondents was found to be 19.3%, mild depression was 23.3%, moderate depression was 39.1%, moderately severe depression was 9.9%, and severe depression was 8.4%, as shown in Figure 1.

### Association between Demographic Characteristics of Health Care Providers and Depression

On bivariate chi-square analysis, half of the sociodemographic aspects demonstrated a significant relationship with depression. Those that were less than 30 years were less likely to be depressed (OR 0.5; 95% CI, 0.3 to 0.8; *P* =.006), and those who were married were more likely to be depressed (OR 3.2; 95% CI, 1.7 to 6.1; *P* <.001), respectively, as shown in table 2.

**Table 2:** Association Between Demographic Characteristics of Health Care Providers and Depression

Sociodemographic characteristics	Depression		OR	95% CI	*P Value*
	Yes	No			
Age					
<=30	47 (48)	51 (52)	0.5	0.3–0.8	.006
>30	69 (66.3)	35 (33.7)			
Gender					
Male	67 (56.3)	52 (43.7)	0.9	0.5–1.6	.405
Female	49 (59)	34 (41)			
Religion					
Christian	112 (57.7)	82 (42.3)	1.4	0.3–5.6.	466
Muslim	4 (50)	4 (50)			
Marital status					
Married	94 (65.7)	49 (34.3)			
Not Married	22 (37.3)	37 (62.7)	3.2	1.7–6.1	<.001

### The Association between Occupational Factors and Depression

From the study, more than 50% of the workers interviewed had less than 6 years of work experience. The mean work experience was 9.3±7.6 SEM=0.5, and this variable was regrouped to a binary variable using the median (6) and the grouping criteria. The majority of the health care providers were nurses. 58.4% (118) and 46.5% (94) of the respondents directly provided COVID-19 care, and 93.1% (188) of the workers had previously been in direct contact with COVID-19 cases. 76.2% (154) had been trained on COVID-19 care. The uptake of the COVID-19 vaccine was at 98% (198), and 42.1% (85) of the staff had previously worked during a pandemic. Workplace precautionary measures were rated as insufficient by 68.3% (138) of the respondents, while 77.2% (156) felt that the personal protective equipment was inadequate. On the nature of work duties, 62.9% (127) had their duties being irregular during the period of the pandemic. 6.4% (13) had been subjected to disciplinary measures during the pandemic period, and the majority, 52.5% (106), had less than six years of experience. These results are summarised in Table 3.

**Table 3:** Occupational Characteristics

Occupational Characteristics	Frequency	Percent
Cadre		
Nurse	118	58.4
Medical doctor	36	17.8
Clinical officer	22	10.9
Laboratory technician	16	7.9
Pharmacist	7	3.5
Dental Officer	3	1.5
Direct COVID-19 patients care		
Yes	94	46.5
No	108	53.5
Years of experience		
<=6	106	52.5
>6	96	47.5
Attended COVID19 training		
Yes	154	76.2
No	48	23.8
COVID19 Vaccinated		
Yes	198	98.0
No	4	2.0
Previous pandemic experience		
Yes	85	42.1
No	117	57.9
Adequacy of workplace precautionary measures.		
Sufficient	64	31.7
Insufficient	138	68.3
Nature of work duties during COVID19 pandemic		
Regular	75	37.1
Irregular	127	62.9
The state of PPEs		
Sufficient	42	20.8
Insufficient	156	77.2
Been subjected to disciplinary measures during the pandemic		
Yes	13	6.4
No	149	73.8
Has been contact of COVID19 patient		
Yes	188	93.1
No	14	6.9

### Association between Occupational Factors and Depression

Those health care providers who had fewer years of work experience and had regular duties during the pandemic had a significantly lower risk of depression (OR 0.5; 95% CI, 0.3 to 0.9; *P* =.018) and (OR 0.5; 95% CI, 0.3 to 0.9; *P* =.013), respectively, while those with previous pandemic experience and those who attended COVID-19 training had a higher risk compared to the rest (OR 1.8; 95% CI, 1 to 3.3; *P* =.027) and (OR 2.7; 95% CI, 1.3 to 5.3; *P* =.004), respectively, as shown in Table 4.

**Table 4:** Association Between Occupational Factors and Depression

Occupational Aspects	Depression		OR	95% CI	*P Value*
	Yes	No			
Nurse vs Other cadres			1	0.6–1.8	.530
Nurse	68 (57.6)	50 (42.4)			
Other cadres	48 (57.1)	36 (42.9)			
Medical doctor vs other cadres			0.9	0.4–1.9	.472
Medical doctor	20 (55.6)	16 (44.4)			
Other cadres	96 (57.8)	70 (42.2)			
Direct COVID-19 patients care			0.9	0.5–1.5	.336
Yes	52 (55.3)	42 (44.7)			
No	64 (59.3)	44 (40.7)			
Years of experience			0.5	0.3–0.9	.018
<=6	53 (50)	53 (50)			
>6	63 (65.6)	33 (34.4)			
COVID19 Vaccinated			1.4	0.2–9.8	.570
Yes	114 (57.6)	84 (42.4)			
No	2 (50)	2 (50)			
Previous pandemic experience			1.8	1–3.3	.027
Yes	56 (65.9)	29 (34.1)			
No	60 (51.3)	57 (48.7)			
Adequacy of workplace precautionary measures.			0.8	0.5–1.5	.350
Sufficient	35 (54.7)	29 (45.3)			
Insufficient	81 (58.7)	57 (41.3)			
Attended C OVID19 training			2.7	1.3–5.3	.004
Yes	99 (62.7)	59 (37.3)			
No	17 (38.6)	27 (61.4)			
Nature of work duties during COVID-19 pandemic			0.5	0.3–0.9	.013
Regular	35 (46.7)	40 (53.3)			
Irregular	81 (63.8)	46 (36.2)			
The state of PPEs			0.6	0.3–1.3	.137
Sufficient	21 (50)	21 (50)			
Insufficient	95 (60.9)	61 (39.1)			
Been subjected to disciplinary measures during the pandemic			0.7	0.2–2.2	.371
Yes	7 (53.8)	6 (46.2)			
No	93 (62.4)	56 (37.6)			
Has been contact of COVID19 patient			1.4	0.5–4.1	.378
Yes	109 (58)	79 (42)			
No	7 (50)	7 (50)			
Relationship with the COVID 19 contact			1.1	0.5–2.4	.529
Family member	16 (59.3)	11 (40.7)			
Client/Patient	93 (57.8)	68 (42.2)			

### Psychological Factors and Depression

Over 94% (191) were worried or fearful during the pandemic; 94% (191) knew a health care provider who contracted the COVID-19 virus, and over 65% (132) received unreliably excessive information during the pandemic, with over 80% reporting the hospital did not have any psychological support for health care providers. Those who faced stigma during the pandemic were 50% (101), over 86% (174) perceived a high-risk level at the workplace, and over 91% (168) rated perceived psychological effects among workmates as high as shown in table 5.

**Table 5:** Psychological Factors of Health Care Providers

Psychological factors	Frequency N=202	Percent %
Has had fear or become worried-working during the pandemic		
Yes	191	94.6
No	11	5.4
Knows a health care worker who-contracted COVID19		
Yes	191	94.6
No	11	5.4
Hospital has psychological support-services for HCW during the pandemic		
Yes	33	16.3
No	162	80.2
Has faced COVID-19-related stigma		
Yes	101	50.0
No	101	50.0
Has received unreliable excessive-amount of information about-COVID 19		
Yes	132	65.3
No	70	34.7
Perception of risk level at the workplace-during the pandemic		
High risk	174	86.1
Low risk	28	13.9
Rating of perceived COVID-19-related psychological effects among-workmates		
High	168	91.8
Low	15	8.2

### Association of Psychological Factors and Depression

Table 6 shows there was a significantly higher occurrence of depression among those who had faced COVID-19-related stigma (OR 2.1; 95% CI, 1.2 to 3.7; *P =.008*). Lower occurrence of depression was demonstrated among those who thought the hospital had psychological support services for health care providers during the pandemic (OR 0.5; 95% CI, 0.2 to 1; *P = 0.043*).

**Table 6:** Association Between Psychological Factors and Depression

Psychological factors	Depression		OR	95% CI	*P Value*
	Yes	No			
Has had fear or become worried working-during the pandemic			0.8	0.2–2.7	.460
Yes	109 (57.1)	82 (42.9)			
No	7 (63.6)	4 (36.4)			
Knows a health care worker who contracted-COVID 19			1.7	0.5–5.6	.302
Yes	111 (58.1)	80 (41.9)			
No	5 (45.5)	6 (54.5)			
Hospital has a psychological support services-for HCW			0.5	0.2 – 1	.043
Yes	14 (42.4)	19 (57.6)			
No	98 (60.5)	64 (39.5)			
Has faced COVID-19 related stigma			2.1	1.2–3.7	.008
Yes	67 (66.3)	34 (33.7)			
No	49 (48.5)	52 (51.5)			
Has received unreliable excessive amount-of information about COVID 19			0.9	0.5–1.7	.465
Yes	75 (56.8)	57 (43.2)			
No	41 (58.6)	29 (41.4)			
Perception of risk level at the work place-during the pandemic			0.9	0.4–1.9	.434
High	99 (56.9)	75 (43.1)			
Low	17 (60.7)	11 (39.3)			
Rating of COVID19 related psychological-effects among workmates			0.5	0.2–1.7	.209
High	99 (58.9)	69 (41.1)			
Low	11 (73.3)	4 (26.7)			

## DISCUSSION

The prevalence of depression in the study was high among health care providers at 57.4%. This was similar to a global study across 31 countries that showed an overall prevalence of 53% for depression.^13^ The workplace environment has effects on the occurrence of depression among health care providers during COVID-19, ^14^ with the current study demonstrating that health care providers who had contact with COVID-19 cases or suspected cases and those who knew a colleague who had contracted COVID-19 had a high likelihood of depression. Similarly, those with previous pandemic experience and those who attended COVID-19 training and had more years of work experience during the COVID-19 pandemic had a higher preponderance for depression.

Those with fewer years of work experience and who had regular duties during the pandemic had a significantly lower risk of depression. These findings are similar to a study that demonstrated the degree of contact with confirmed or suspected cases of COVID-19 was directly proportional to stress levels among health care providers.^15^ On COVID-19 training, our study failed to concur with a study during the SARS epidemic that demonstrated those who had received training were less likely to suffer psychological effects. ^16^ Health care providers who received inadequate training related to the management of the pandemic had higher psychological morbidity than those who had been given appropriate training.^17^

A study during the pandemic demonstrated that most of the staff mentioned that they did not need a psychologist but more rest, a regular work schedule, and adequate personal protective equipment. They suggested training on psychological skills to deal with patients’ psychological responses to COVID-19 infection and requested for a mental health staff to be incorporated in direct care. ^18^

A recommendation for sustained exposure to emergency preparation through training, performing emergency drills, and educational sessions by the various disaster and emergency teams of the hospital.^19^ This will improve the health care providers’ psychological resilience during the pandemic. The World Health Organization (WHO) has made a call to governments to provide health care providers with better protection, which includes access to vaccines, personal protective equipment, training, psychological support, decent working conditions, including adequate remuneration, and protection against excessive workloads.

This study demonstrated reduced depression among those who thought that the hospital had better psychological support services for the health care providers during the COVID-19 pandemic. The study findings concurred with a study that observed that psychological interventions, preferably delivered over the telephone, were shown to be helpful in reducing psychological responses. ^20^ The perception that the hospital had adequate psychological support to assure psychological resilience of workers reduced the occurrence of mental health-related problems among health care providers. One of the studies went ahead and detailed the telephone-based psychological support for frontline workers in the initial COVID-19 outbreak in Wuhan and how the calls and debriefing sessions went a long way in enhancing the resilience of the workers when there was still high uncertainty about the trajectory, care, and treatment of the cases of the novel agent.^21,22^

The current study looked into the excessive, unreliable amount of information about COVID-19 and its association with health care providers’ psychological responses. Though there was no significant association between those who received the information and those who did not receive it, it is worth noting that the majority reported receiving a lot of unreliable information (132). Receiving unreliable information and falsified reports about COVID-19 leads to misinformation, which can exacerbate depressive symptoms. Thus, it is imperative to update and get accurate information, especially on the number of recoveries, as this is associated with lower depressive symptoms during COVID-19.^23^ Likewise, several studies demonstrated that psychological shock from overwhelming information emerging about the disease made worse the feelings of pessimism about the trajectory of the disease and caused a post-traumatic stress-like response among medical staff. Younger people tend to obtain large amounts of information from social media, triggering stress, and people with higher education tended to have more distress, probably because of high self-awareness of their health and increased risk perception.^24,25^ On the contrary, a study showed providing accurate and timely information to health care providers about Severe Acute Respiratory Syndrome (SARS) reduced stigma related to care and contracting of the disease.^26^

This study’s findings indicate a high likelihood of depression among those who faced stigma. This is similar to findings where many healthcare workers in the recent Ebola and SARS epidemics experienced considerable stigmatisation, loneliness, and even loss of trust within their own communities.^26^ Stigma towards those caring for COVID-19 and those who contracted the disease was quite high across the globe but more specifically in the countries that had the most severe outcomes of the disease, like Italy. Some studies demonstrated that risk perception at the workplace led to more negative psychological effects of social stigma related to fatality and high transmissibility of the disease, and some health care providers feared role reversal from care provider to patient and the attendant stigma of the COVID-19 sick role. ^28,29^

Family and friends’ support for health care providers during COVID-19 was rated as very important, especially when facing stigma from the community. Job-related considerations like sick leave and telephone psychological care encouraged resilience towards the effects of stigma. ^30^ Most of the health care providers had concerns over contracting the disease and transmitting it to family members and the community, stigmatising them for that and due to providing COVID-19 care. However, hero campaigns for health care workers by the government and other agencies were shown to alleviate the effects of stigma and equally reduce the stigma towards them and their families. ^31^

Workplace high-risk perception and rating of COVID-19-related psychological effects among colleagues increased depression among health care providers, as demonstrated by the current study. This is in agreement with Italian studies that showed that risk perception was directly proportional to stress level among health care workers and that the frontline line caregivers were the ones at most risk.^32,33^ A lower risk perception was associated with less severe acute respiratory syndrome-related stress among health care providers. However, balanced risk perception is key in encouraging preventive measures like handwashing and use of personal protective equipment.^34^ Thus, authorities should maximise effective risk communication to optimise perception through helpful evolution of health care providers understanding of the disease and individual risk.

### Study Limitation

This study used an online survey, which posed an increased risk of non-response. The risk was minimised through sending constant reminders and follow-ups to the respondents. The nature of the study may have also introduced a selection bias where non-responders could the health care providers who lacked internet access but with different characteristics compared with the health care providers who responded. This was a cross-sectional design whose data cannot be used to establish causal relationship. The results should therefore be interpreted with this limitation in mind.

## CONCLUSION

The study revealed high rates of significant depression (moderate depression 39.1%, moderately severe depression 9.9%, and severe depression 8.4%). Occupational factors like direct patient care, fewer years of experience, sufficient personal protective equipment, and other supplies led to reduced levels of depression. Stigma towards health care providers who contracted or cared for COVID-19 patients increased the vulnerability of having depression.
